# Interactive Versus Static Decision Support Tools for COVID-19: Randomized Controlled Trial

**DOI:** 10.2196/33733

**Published:** 2022-04-15

**Authors:** Alice Röbbelen, Malte L Schmieding, Marvin Kopka, Felix Balzer, Markus A Feufel

**Affiliations:** 1 Division of Ergonomics Department of Psychology and Ergonomics (IPA) Technische Universität Berlin Berlin Germany; 2 Institute of Medical Informatics Charité – Universitätsmedizin Berlin Corporate Member of Freie Universität Berlin and Humboldt-Universität zu Berlin Berlin Germany; 3 Cognitive Psychology and Ergonomics Department of Psychology and Ergonomics (IPA) Technische Universität Berlin Berlin Germany

**Keywords:** clinical decision support, usability, COVID-19, consumer health, medical informatic, symptom checker, decision support, symptom, support, decision making, algorithm, flowchart, agent

## Abstract

**Background:**

During the COVID-19 pandemic, medical laypersons with symptoms indicative of a COVID-19 infection commonly sought guidance on whether and where to find medical care. Numerous web-based decision support tools (DSTs) have been developed, both by public and commercial stakeholders, to assist their decision making. Though most of the DSTs’ underlying algorithms are similar and simple decision trees, their mode of presentation differs: some DSTs present a static flowchart, while others are designed as a conversational agent, guiding the user through the decision tree’s nodes step-by-step in an interactive manner.

**Objective:**

This study aims to investigate whether interactive DSTs provide greater decision support than noninteractive (ie, static) flowcharts.

**Methods:**

We developed mock interfaces for 2 DSTs (1 static, 1 interactive), mimicking patient-facing, freely available DSTs for COVID-19-related self-assessment. Their underlying algorithm was identical and based on the Centers for Disease Control and Prevention’s guidelines. We recruited adult US residents online in November 2020. Participants appraised the appropriate social and care-seeking behavior for 7 fictitious descriptions of patients (case vignettes). Participants in the experimental groups received either the static or the interactive mock DST as support, while the control group appraised the case vignettes unsupported. We determined participants’ accuracy, decision certainty (after deciding), and mental effort to measure the quality of decision support. Participants’ ratings of the DSTs’ usefulness, ease of use, trust, and future intention to use the tools served as measures to analyze differences in participants’ perception of the tools. We used ANOVAs and *t* tests to assess statistical significance.

**Results:**

Our survey yielded 196 responses. The mean number of correct assessments was higher in the intervention groups (interactive DST group: mean 11.71, SD 2.37; static DST group: mean 11.45, SD 2.48) than in the control group (mean 10.17, SD 2.00). Decisional certainty was significantly higher in the experimental groups (interactive DST group: mean 80.7%, SD 14.1%; static DST group: mean 80.5%, SD 15.8%) compared to the control group (mean 65.8%, SD 20.8%). The differences in these measures proved statistically significant in *t* tests comparing each intervention group with the control group (*P*<.001 for all 4 *t* tests). ANOVA detected no significant differences regarding mental effort between the 3 study groups. Differences between the 2 intervention groups were of small effect sizes and nonsignificant for all 3 measures of the quality of decision support and most measures of participants’ perception of the DSTs.

**Conclusions:**

When the decision space is limited, as is the case in common COVID-19 self-assessment DSTs, static flowcharts might prove as beneficial in enhancing decision quality as interactive tools. Given that static flowcharts reveal the underlying decision algorithm more transparently and require less effort to develop, they might prove more efficient in providing guidance to the public. Further research should validate our findings on different use cases, elaborate on the trade-off between transparency and convenience in DSTs, and investigate whether subgroups of users benefit more with 1 type of user interface than the other.

**Trial Registration:**

Deutsches Register Klinischer Studien DRKS00028136; https://tinyurl.com/4bcfausx (retrospectively registered)

## Introduction

In December 2019, the spread of the novel lung disease COVID-19 caused by SARS-CoV-2 with previously unknown etiology was detected, and it developed into a global pandemic within a few weeks [1,2]. The disease courses of COVID-19 are heterogeneous. On the one hand, it is dangerous and can be lethal, even among previously healthy individuals. On the other hand, COVID-19 can present itself with unspecific and mild symptoms [3]. Given the dynamic development of the disease and public health measures, laypersons often felt uncertain about appropriate behavior, especially when a COVID-19 infection was suspected but not proven. This situation resulted in considerable uncertainty both about what level of medical care was needed (care-seeking behavior) and whether isolation or quarantine was required (social behavior). As a result, capacity utilization in the health care system increased [4].

The increased demand for medical advice could not be met through traditional routes (eg, telephone consultation, visit to the general practitioner, visit to the emergency room, local health authorities). As a consequence, various online services with information and self-assessments (ie, patient-facing clinical decision support tools [DSTs]) were developed. These DSTs are comparable to commonly known symptom checkers (SCs), which “are tools developed to provide support to laypersons. Users can enter their complaints and, with some SCs, demographic or health-related information (eg, age, gender, past medical history) to obtain advice on the urgency of their complaints (triage advice) and the most likely diagnosis” [5]. The DSTs considered in the context of COVID-19 often require information about possible exposure to the virus, in addition to standard inputs related to gender, age, and major complaints. As output, the DSTs provide the probability of a COVID-19 infection rather than a diagnosis. Though most of the underlying algorithms are rule-based, simple, and similar decision trees, implementing official guidelines (eg, of the Centers for Disease Control and Prevention [CDC] [6]), their mode of presentation differs: Some DSTs present the rule-based decision tree algorithm as a static flowchart [7-12], while others are designed as conversational agents (ie, similar to chatbots) with a graphical user interface, which guides the user through the decision tree’s nodes step-by-step in an interactive manner [13-21].

Our review of the literature suggests that there is no research, neither on SCs nor on DSTs for COVID-19, indicating whether and how the interactivity of a user interface influences the decision outcome and decision support experience. Currently available studies assessing DSTs for COVID-19 report no findings on the influence between user interaction and quality of decision support [4,22-26]. However, many publications on so-called patient decision aids (PtDAs) exist that have examined web-based and paper-based decision aids. In these studies, web-based PtDAs showed no difference from paper-based PtDAs in their effect on participants’ decision making [27]. In terms of web-based PtDAs, interactive and static formats have not yet been compared to the best of our knowledge.

However, the comparison between web-based tools is particularly relevant during the COVID-19 pandemic, as laypersons’ decision making should ideally be supported at home to minimize the risks of unnecessary COVID-19 infections. The aim of this study is to assess whether static and interactive DSTs increase laypersons’ accuracy and confidence when making COVID-19-related decisions via the internet and whether 1 type of DST (static or interactive) is superior to the other.

## Methods

### Study Design

We chose a between-subjects design with 1 three-level independent variable: participants had to solve fictional case vignettes while receiving different types of decision support (no support in the control group, a DST in the form of a static flowchart in the first intervention group, and an interactive DST in the second intervention group). Participants were randomly assigned to 1 of the 3 groups. Decision support quality was evaluated by analyzing the tools’ effects on decision making and the participants’ judgement of the tools with multiple dependent variables: Regarding effects on decision making, the number of correct appraisals (using official CDC recommendations from October 14, 2020, as the standard [28]), perceived certainty regarding the appraisal, and mental effort in making the appraisals were examined. Participants' judgments were collected on tool usefulness, ease of use, trust in the tools’ recommendations, and intention to use the tools in the future.

### Ethics Approval and Consent to Participate

The Ethics Committee of the Department of Psychology and Ergonomics (IPA) at the Technische Universität Berlin approved this study (tracking number ROEB_01_20200715). Participants volunteered to take part in the survey and provided informed consent before participating.

### Development of the Decision Support Tools

We designed 2 mock DSTs. The design was intended to mimic existing tools available on the internet. Features of various tools related to COVID-19 were analyzed. Relevant common features of these tools were the use of short, precise questions or statements to which the participants could respond in agreement (yes) or disagreement (no), and sometimes also with uncertainty (unsure). These questions guided participants through a decision tree’s nodes step-by-step until a recommendation could be provided. Input requirements and output content were mostly based on recommendations from public health agencies, such as the CDC. For the purpose of our study, the John Hopkins University Coronavirus Self-Checker was identified as a most suitable template because it is a decision tree–based interactive tool that requires a small amount of information at each step as input [18]. This facilitated the construction of a second DST providing identical advice in the form of a static flowchart, outlining the entire decision tree at once. In both cases, the content was based on the CDC recommendations from October 14, 2020 [28].

Considering these aspects, we developed 2 mock DSTs with identical content that differed only with respect to the interactivity of their user interface: a static flowchart where participants could follow the arrows of the appropriate path using their gaze (Multimedia Appendix 1). This flowchart was developed using Microsoft PowerPoint [29]. The other tool was implemented as a simple, interactive conversational agent, where participants clicked buttons to respond to questions, guiding them through the nodes of the decision tree step-by-step (Multimedia Appendix 2). The individual screens of the interactive DST were also designed using Microsoft PowerPoint and then linked together using InVision [30], enabling dynamic interaction. Mimicking the output of existing tools, 5 different recommendations were possible: “emergency care is required,” “self-care and physical distancing are sufficient,” “self-care is sufficient and quarantine necessary,” “self-care is sufficient and isolation is necessary,” and “nonemergency care and isolation are required.” We anticipated that laypersons would struggle comprehending the difference between “quarantine” and “isolation,” yet we decided to differentiate between them, as some COVID-19 DSTs use these terms without explaining them [7,11]. During data analyses, however, we rated answers as correct when participants confused these 2 terms (see the Data Analysis section). We developed mock tools rather than choosing existing DSTs from the internet in order to ensure comparability of the algorithm and design of the tools, as well as stable integration into the survey throughout the surveying period.

### Preparing the Case Vignettes

We created 7 short patient descriptions (case vignettes). In this process, we extracted decision criteria identified by the CDC and its recommendations concerning help-seeking and social behavior in the case of a potential COVID-19 infection [28]. Decision criteria included the presence of typical COVID-19 symptoms with the following expressions: 0, no symptoms; 1, primary symptoms (typical for a COVID-19 infection); and 2, secondary symptoms (can also occur in a COVID-19 infection but are less typical) (see Multimedia Appendix 3) [28]. Another decision criterion represented the potential contact with the pathogen SARS-CoV-2 and the following expressions: 1, critical contact with a confirmed infected person has occurred; 2, contact may have occurred; or 3, critical contact can be excluded. The last important criterion is the presence or absence of risk factors classified by the Robert Koch Institute (RKI), Germany’s federal public health authority (eg, certain pre-existing conditions such as chronic lung disease; see Multimedia Appendix 4) [3]. Through a pretest with 15 participants, in which we evaluated the response behavior of the participants as well as their free-text feedback, we revised the wording of the vignettes, improving intelligibility and removing errors spotted by the pretest participants. The vignettes included more information than necessary for the appraisal to better simulate a real-life decisional context and increase ecological validity. We also ensured that all information asked for in the developed DSTs was included in the vignettes and that the fictitious patients represented diverse age and gender groups. In addition, each possible outcome of the mock tools was covered at least once as a correct solution. Multimedia Appendices 5 and 6 contain an outline of the 7 case vignettes.

### Data Collection

#### Participants

All participants lived in the United States, were at least 18 years old, and had no professional medical background. Our investigation was limited to US residents, as recommendations of the developed DSTs were based on CDC recommendations and guidelines and thus applied to the US region.

#### Survey and Instruments

We created an online survey using UNIPARK (QuestBack GmbH, Germany) [31]. To ensure differences between the 2 groups were not due to confounding variables, we surveyed variables we suspected to have an influence on the main outcome measures, namely sociodemographic factors, affinity toward interacting with technology, prior knowledge of COVID-19, prior use of a COVID-19 DST, and perceived threat of a COVID-19 infection. The sociodemographic variables were age, gender, US residency, and highest level of completed formal education. Affinity toward interacting with technology was surveyed with the Affinity for Technology Interaction Short Scale (ATI-S) [32]. The perceived threat of COVID-19 was surveyed with an instrument developed by Kim and Park [33]. Participants’ prior knowledge of COVID-19 necessary to appraise the adequate health-seeking and social behavior for a suspected COVID-19 patient was assessed with 5 self-developed multiple-choice questions (see Multimedia Appendix 7).

In the survey’s main part, participants were presented the 7 case vignettes in random order. In both the experimental groups and the control group, participants provided their personal appraisal of the adequate help-seeking behavior for each case vignette and, in a further question, of the adequate social behavior. In the 2 experimental groups, participants were also prompted to state which recommendations of the tool (supposedly) provided concerned help-seeking behavior and social behavior (see Multimedia Appendices 8 and 9).

As we anticipated participants commonly erring in determining the appropriate social behavior when required to differentiate between isolation and quarantine, we deemed both answers correct for our main analysis and conducted a second analysis without this adjustment (Multimedia Appendix 9).

After each case vignette, participants were further asked to rate their mental effort required in making decisions concerning the respective fictitious case presentation using a 9-point category scale ranging from very, very low mental effort to very, very high mental effort (see Multimedia Appendix 10) [34]. This scale by Paas et al [35] can be considered a “subjective, indirect measure of cognitive load” in making decisions regarding case vignettes. Since mental effort increases with higher perceived demands of a stimulus or task [35], it was included here as an indicator of the quality of decision support. We assume that a good DST guides people to the right decision without requiring high cognitive load.

Following all 7 case vignettes, participants’ decisional uncertainty was assessed once using the O'Connor [36] Decisional Conflict Subscale. On 3 items with a 5-point scale, participants rated how confident they were in their decisions (see Multimedia Appendix 11). The scale was developed to evaluate health care consumer decision aids. We used this scale as an indicator of the quality of decision support, since we assume that a good DST helps medical laypersons to feel confident in their decisions. This assumption is supported by O'Connor's [36] claim that “decision aids should reduce uncertainty and confusion in choosing a course of action.”

In addition to the effect of the tools on decision making and participants' perceptions of their decision making, we also asked directly about participant's perceptions of the tools in the 2 intervention groups. They were asked about the perceived usefulness and ease of use of the tools after having worked on all 7 cases. Perceived usefulness is defined as the individual's perception of the extent to which using the tool improves decision-making performance. Perceived ease of use implies that using the tool does not require any effort [37]. Both constructs were measured using scales from the Davis Technology Acceptance Model [38]. In addition, the intentions to use the DSTs in the future and the trust in the recommendations of the DSTs were measured as a subjective rating using 1 item each (Multimedia Appendix 12 presents the exact phrasing of the items).

#### Procedure

We recruited participants via the crowdsourcing platform Prolific.co [39]. This platform is aimed at researchers and allows them to conveniently recruit participants for online surveys and experiments. Prolific.co provides a pool of participants that can be screened based on demographic data. Researchers can make their survey or experiment available on Prolific.co, and users registered on Prolific.co as prospective participants can choose whether to participate in studies that they are eligible for according to the set prescreening criteria. We chose Prolific.co as it is characterized by a diverse population that provides high-quality data [40]. Each participant received a remuneration of US $2.00 for completing the survey. In addition, participants could earn a bonus of US $0.20 for each correct decision. To ensure that all participants answered the questions attentively, attention checks were added to the survey. Participants failing more than 1 of 3 attention checks were excluded. Furthermore, at the end of the survey, participants were asked to self-report whether they had participated in the survey attentively, honestly, and without external assistance, as suggested by Rouse [41]. Participants were remunerated independently of their answer, but only data from participants confirming this question were included in the analysis. By selecting the weekend day and early afternoon PDTs, we attempted to recruit a population as diverse as possible, as suggested by Casey et al [42]. The survey was released for participation on Prolific.co on 4 different days (November 21, 2020, at 1 PM Pacific Daylight Time [PDT]; November 22, 2020, at 11 AM PDT; November 28, 2020, at 12 PM PDT; and November 29, 2020, at 1 PM PDT). On each day, 50 participants were recruited within a few hours after release.

### Data Analysis

Data were cleaned and explored using R 4.0.2 [43] and the *tidyverse* packages [44]. After examining the central tendencies and distributions of the variables separately for the 3 groups and plotting them using the *ggplot2* package [45], we performed a one-way analysis of variance to compare the 3 conditions separately for the dependent measures accuracy, decisional certainty, and mental effort. If significant group differences were present, we followed up with Bonferroni-corrected post hoc pairwise *t* tests. For the dependent variables’ usefulness, ease of use, trust in tool recommendations, and future use intention, we conducted Welch two-sample *t* tests to compare the 2 intervention groups because the sample sizes of the groups were unequal [46]. Effect sizes were quantified with Cohen *d* and calculated using the *rstatix* package [47]. When results were not statistically significant, we performed a post hoc power analysis using the *pwr* package [48] with corresponding group sizes, an α level of .05, and a power 1-β of .80.

## Results

### Participant Characteristics

In total, our survey was accessed 233 times during the 4 days it was available. Overall, 37 participations could not be used for data analysis because they dropped out of the questionnaire (n=12, 32%), exceeded the maximum survey completion time (n=10, 27%), failed attention checks (n=6, 16%), or did not meet eligibility criteria (n=9, 25%). The remaining participants all affirmed that they had paid close attention during the survey and answered honestly. This yielded a total of 196 (84.1%) participants, who assessed all 7 case vignettes and could be included in the analysis (see Table 1 for details). The mean time for completion of the survey was 22 minutes and 3 seconds. Across the 3 groups, the participant characteristics were similar; see Table 1.

**Table 1:** Participant characteristics (N=196) of an experimental study assessing the influence of DSTs^a^ on laypersons’ COVID-19-related appraisals. Participants were nonmedically trained US inhabitants sampled online in November 2020.

Characteristics			Total sample		Group 1: control group (no DST)		Group 2: static DST		Group 3: interactive DST
Sample size, N			196		66		62		68
Age (years), median (IQR)			30 (18)		30 (17.2)		26.5 (13.2)		33 (20.5)
**Gender, n (%)**									
	Female	94 (48)		31 (47)		27 (44)		36 (53)	
	Male	100 (51)		33 (50)		35 (56)		32 (47)	
	Other	2 (1)		2 (3)		0		0	
**Education, n (%)**									
	Non-high-school graduate	4 (2)		1 (2)		1 (2)		2 (3)	
	High school graduate	34 (17)		9 (14)		18 (29)		7 (10)	
	Some college	66 (34)		22 (33)		20 (32)		24 (35)	
	Bachelor’s degree	49 (25)		20 (30)		12 (19)		17 (25)	
	Graduate degree or higher	43 (22)		14 (21)		11 (18)		18 (26)	
**Recent^b^ experience with COVID-19 decisions, n (%)**									
	Recently faced a triage decision	70 (35)		25 (38)		27 (44)		18 (26)	
	Recently faced a social behavior decision	101 (52)		35 (53)		32 (52)		34 (50)	
	Recently consulted a static DST to face the decision	56 (29)		15 (23)		21 (34)		20 (29)	
	Recently consulted an interactive DST to face the decision	53 (27)		21 (32)		15 (24)		17 (25)	
**Medical training, n (%)**									
	No training	163 (83)		55 (83)		51 (82)		57 (84)	
	Basic first-aid training	33 (17)		11 (17)		11 (18)		11 (16)	
Affinity for technology interaction on a scale of 1-6, median (IQR)^c^			4 (1.2)		4 (1.7)		4 (1)		4 (1)
Perceived threat of COVID-19 on a scale of 1-7, median (IQR)^d^			5 (2)		5.25 (1.6)		5 (2.1)		5 (2)
Prior knowledge of COVID-19 on a scale of 0-5, median (IQR)^e^			3 (2)		3 (2)		3 (1)		3 (1)

^a^DST: decision support tool.

^b^“Recent” was defined as “in the past 6 months.”

^c^Measured by the Wessel Affinity for Technology Interaction Short Scale (ATI-S).

^d^ Measured by a subjective self-assessment on 2 items on a scale of 1-7 adapted from Kim and Park [33].

^e^Measured by the number of correctly answered multiple-choice questions with reference to COVID-19.

### Effects on Decision Making

Omnibus ANOVAs detected group differences with respect to decision accuracy (overall and separately for decisions about help-seeking behavior and social behavior) and perceived certainty but not for mental effort in decision making; see Table 2.

**Table 2:** Omnibus ANOVAs measuring the effects of different DSTs^a^ on laypersons’ ability to correctly appraise fictitious descriptions of patients with symptoms indicative of COVID-19 in an experimental study. In total, 196 participants (all US residents and nonmedically trained) were recruited online in November 2020 and asked to judge how fictitious patients with symptoms indicative of COVID-19 should behave. Participants were randomly assigned to 1 of 3 groups in which they either received support by a static DST (flowchart) or an interactive DST (mimicking a conversational agent) or received no support.

Dependent variables		Group 1: without DST		Group 2: static DST		Group 3: interactive DST		Test statistics of group comparison				
								*F*_2,193_		*P* value		η²
**Accuracy, mean (SD)**												
	Total correct decisions (min=0, max=14)^b^	10.17 (2.00)	11.45 (2.48)		11.71 (2.37)		8.59		<.001		0.08	
	Correct decisions on help-seeking behavior (min=0, max=7)	4.82 (0.96)	5.47 (1.39)		5.54 (1.40)		6.58		<.001		0.002	
	Correct decisions on social behavior (min=0, max=7)^b^	5.35 (1.41)	5.98 (1.26)		6.16 (1.18)		7.33		<.001		0.02	
Decisional certainty: certainty score from 0 to 100^c^, mean (SD)		65.78 (20.78)		80.51 (15.89)		80.72 (14.08)		15.67		<.001		0.14
Mental effort on a scale from 1 to 9, mean (SD)		5.62 (1.57)		5.40 (1.68)		5.09 (1.78)		1.73		.18		N/A^d^

^a^DST: decision support tool.

^b^The response options “quarantine” and “isolation” were not differentiated for this analysis, that is, they were both considered correct because layperson participants commonly confuse these terms. An analysis without this adjustment is provided in Multimedia Appendix 9 and shows the same trend.

^c^Responses were transformed into a certainty score between 0 and 100 (ie, 0=person feels extremely uncertain about the best choice and 100=person feels extremely certain about the best choice).

^d^N/A: not applicable.

#### Accuracy

**Figure 1:**
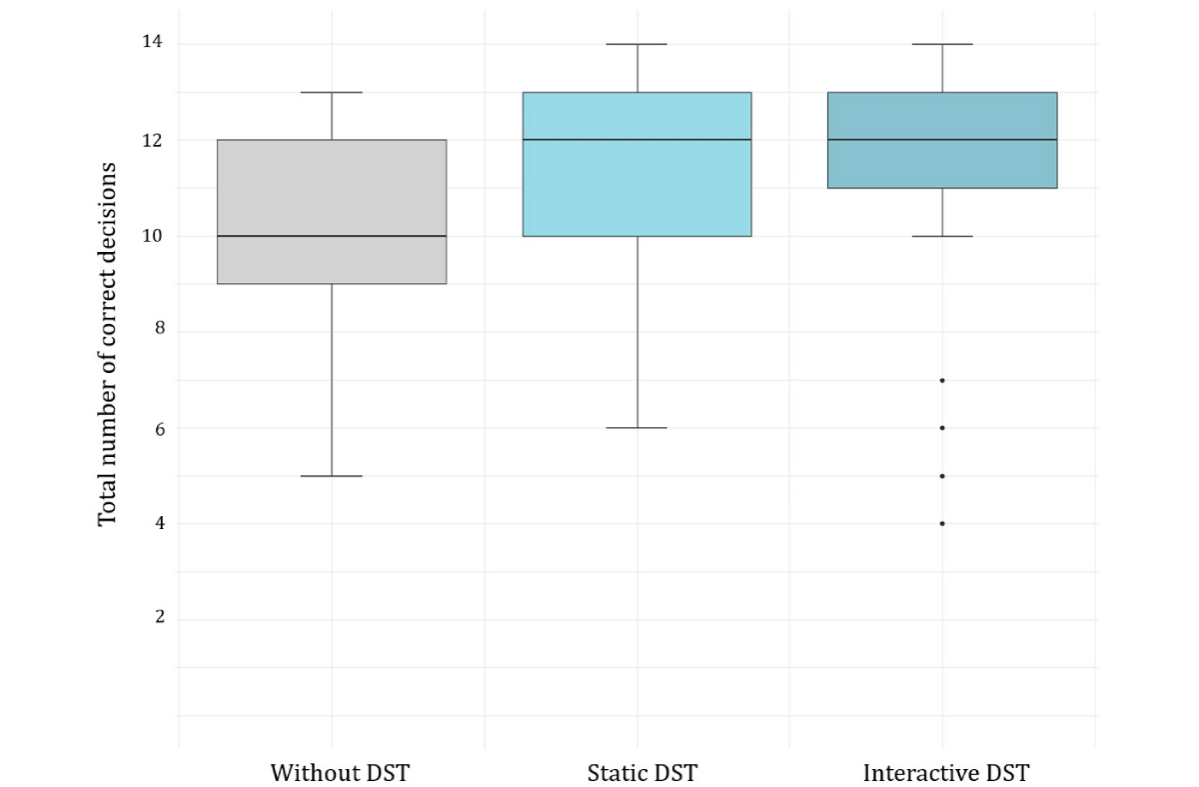
Boxplot showing the distribution of the 196 participants’ decision accuracy to appraise 7 fictitious descriptions of patients with symptoms indicative of COVID-19. Study participants (all US inhabitants, nonmedically trained, sampled online in November 2020) were tasked to answer 2 questions per patient description. We randomly assigned participants to 1 of 3 experimental groups; in 2 groups, they were supported by either a static DST (ie, a flowchart) or an interactive DST (ie, a conversational agent mimicking a chatbot). In the control group, they received no decision support. The boxplots’ filled box represents the IQR, the horizontal line inside the box the median, the whiskers the maximum and minimum values within 1.5 IQR of the median, and the single dots the outliers of participants’ total number of correct decisions. DST: decision support tool.

Across all groups, participants’ appraisals were commonly correct. The average participant decided correctly in more than 10 (70%) of the 14 decisions. Overall decision accuracy was higher in the experimental groups receiving support from a DST than in the unsupported control group; see Figure 1 and Table 2. Bonferroni-corrected pairwise Welch two-sample *t* tests indicated statistically significant differences with moderate effect sizes between control and experimental groups (control group vs static DST group: t_117.35_=–3.21, *P*<.001, Cohen *d*=–0.57, 95% CI –1.1 to –0.14; control group vs interactive DST group: t_129.6_=–4.06, *P*<.001, Cohen *d*=–0.70, 95% CI –1.31 to –0.21). The difference in decision accuracy between the 2 experimental groups showed a negligible effect size and was not significant (t_125.58_=–0.59, *P*=.55, Cohen *d*=–0.10, 95% CI –0.54 to 0.34). Based on a post hoc power analysis, we estimated that on a population level, the real difference in accuracy was less than Cohen *d*=0.5 (equivalent to 1.22 correct responses), with a power of 0.8.

We found similar results when analyzing participants’ accuracy for help-seeking behavior and social behavior separately; see Table 2. When the differences between the response options “isolation” and “quarantine” were considered, the pattern remained the same, but the gap in accuracy between experimental and control groups widened; see Multimedia Appendix 13.

#### Decisional Certainty

**Figure 2:**
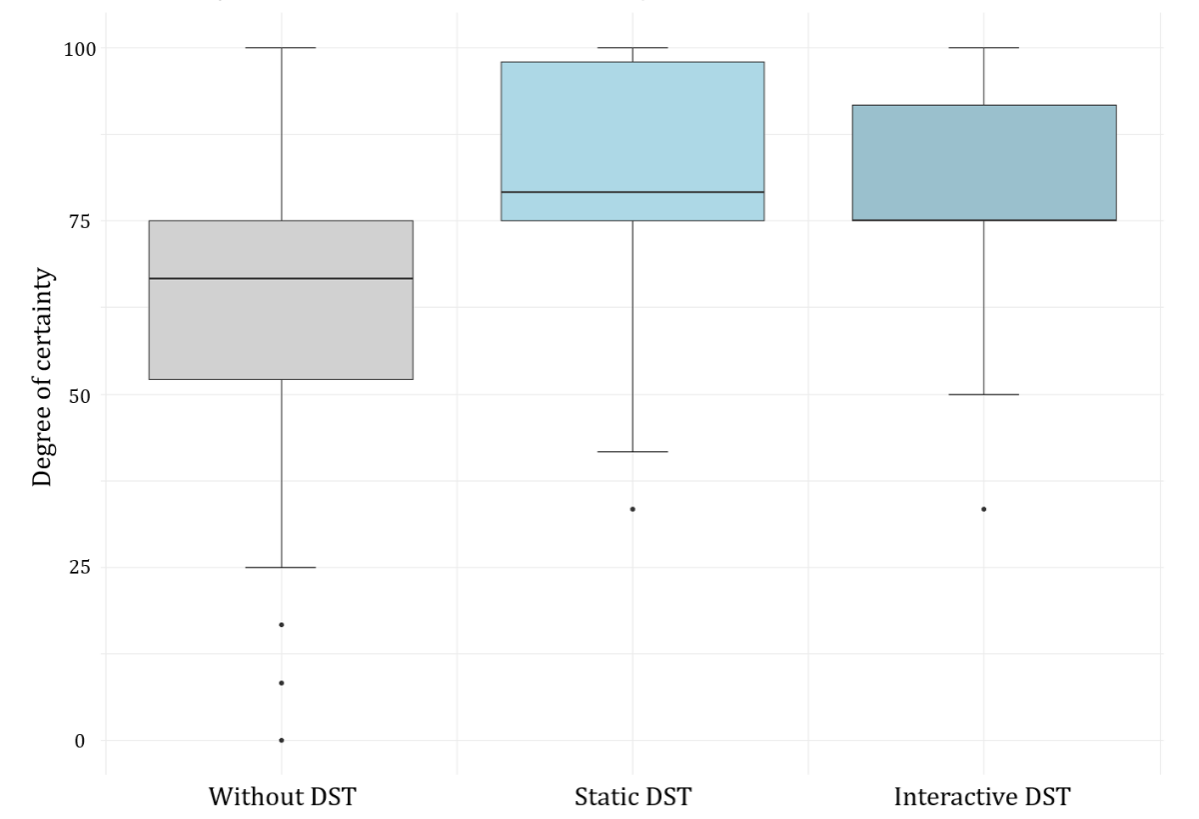
Laypersons’ perceived certainty in their own appraisals of COVID-19-related clinical decisions obtained in an experimental study in November 2020. The 196 study participants were US residents, nonmedically trained, and sampled online. Our study tasked them to assess 7 fictitious descriptions of patients with symptoms indicative of COVID-19. Participants were randomized to either receive support from a static DST (ie, a flowchart) or an interactive DST (ie, a conversational agent mimicking a chatbot). Following the 14 appraisals, we surveyed the participants’ certainty in their answers using the Decisional Conflict Scale. A score of 0% indicated minimum certainty, while 100% indicated maximum certainty. The boxplots’ filled box represents the IQR, the horizontal line inside the box the median, the whiskers the maximum and minimum values within 1.5 IQR of the median, and the single dots the outliers of participants’ total number of correct decisions. DST: decision support tool.

Participants were commonly certain in their decision making. Less than 15 (8%) of the 196 participants indicated a certainty of less than 50 on a scale from 0 to 100, in contrast to 32 (16%) indicating maximum certainty (100/100). Participants’ certainty in their decisions differed between the 3 groups: certainty in decision making was rated lower by participants without decision support than by those receiving decision support; see Figure 2 and Table 2. Bonferroni-corrected pairwise Welch two-sample *t* tests marked these differences as statistically significant with large effect sizes (control group vs static DST group: t_121.1_=–4.51, *P*<.001, Cohen *d*=–0.79, 95% CI –1.22 to –0.37; control group vs interactive DST group: t_115.67_=–4.67, *P*<.001, Cohen *d*=–0.81, 95% CI –1.27 to –0.44).

Decision certainty in the 2 experimental groups was nearly identical, with mean certainty scores of 80.51 (static DST) and 80.72 (interactive DST). The inferential analysis indicated this difference to be of negligible effect size and nonsignificant (t_123.68_=–0.09, *P*=.92, Cohen *d*=0.01, 95% CI –0.47 to 0.43). Based on a post hoc power analysis, we estimated that on a population level, the real difference in decisional certainty between both experimental groups was less than Cohen *d*=0.5 (equivalent to 7.5 percentage points), with a power of 0.8.

#### Mental Effort

The perception of how demanding the task was varied among participants. Although a third (63/196, 32.1%) stated that the tasks required low mental effort (ie, they indicated an average mental effort score below 4.5 on a scale from 1 to 9), 4 (40%) of 10 considered the tasks to be at least somewhat demanding (indicating scores above 5.5).

Participants rated the mental effort of decision making lowest in the interactive DST group (mean 5.06) and highest in the control group unsupported by a DST (mean 5.62); see Table 2. However, the differences between group means were not significant in the omnibus ANOVA; see Table 2. Based on a post hoc power analysis, we estimated that on a population level, the effect size of the difference was not greater than η²=0.047, with a power of 0.8.

### Participants’ Perceptions of the Tools

We investigated participants’ perceptions of the mock tools with 4 metrics: perceived usefulness, perceived ease of use, trust, and intention to use the tools again in the future. On average, participants perceived the DSTs positively: their self-reported trust in the tools was generally high in both experimental groups as was the number of DST recommendations the participants followed (on average, in more than 12 [86%] of a total of 14 recommendations); see Table 3. Accordingly, almost all (126/130, 95%) participants judged the mock tools to be useful (ie, indicated a perceived usefulness score of above 3.0 on a scale from 1 to 5), with half of the participants scoring the usefulness at or above 4.75. Ease of use was rated similarly positive (see Table 3), and their intention to use the tools in the future was high, on average. Regarding differences between the 2 DSTs, the mock users’ scores on perceived usefulness, trust, and future use intention were higher in the static than in the interactive DST group. Ease of use was perceived higher in the interactive DST group. However, all effect sizes of differences between the 2 DST groups were small (Cohen *d*≤0.35) and proved significant only for future use intention in a Welch two-sample *t* test; see Table 3.

Based on a post hoc power analysis, we estimated that on a population level, the real difference in these variables between both groups was less than Cohen *d*=0.5 (equivalent to a difference of 0.34 for perceived usefulness and perceived ease of use, 0.42 for self-reported trust, 0.96 for followed decisions, and 0.51 for future use intention), with a power of 0.8.

**Table 3:** Laypersons’ perceptions of 2 mock DSTs^a^ for COVID-19-related clinical decisions obtained in an experimental study in November 2020. The 196 study participants were US residents, nonmedically trained, and sampled online. Our study tasked them to assess 7 fictitious descriptions of patients with symptoms indicative of COVID-19. Participants were randomized to either receive support from a static DST (ie, a flowchart) or an interactive DST (ie, a conversational agent mimicking a chatbot) or receive no support (control group). Subsequently, participants in the intervention groups were asked to rate the given tools’ usefulness and perceived ease of use, state their trust in the tools, and state their future intention to use the tools. We measured usefulness and perceived ease of use according to the Davis Technology Acceptance Model.

Dependent variables		Group 2: static DST	Group 3: interactive DST	Test statistics of group comparison		
				*t*_df_	*P* value	Cohen *d*
Perceived usefulness on a scale from 1 to 5, mean (SD)		4.56 (0.65)	4.35 (0.71)	t_128_=1.7	.09	0.30
Perceived ease of use on a scale from 1 to 5, mean (SD)		4.26 (0.71)	4.47 (0.58)	t_117.92_=–1.90	.06	–0.34
**Trust, mean (SD)**						
	Self-reported trust in the tools' recommendation on a scale from 1 to 7	6.08 (0.84)	5.83 (0.85)	t_127.15_=1.74	.08	0.31
	Decisions where the recommendation of the tools was followed on a scale from 0 to 14	12.73 (1.81)	12.5 (2.03)	t_127.94_=0.67	.75	0.12
Future use intention on a scale from 1 to 7, mean (SD)		6.23 (0.88)	5.87 (1.16)	t_123.91_=1.99	.05	0.35

^a^DST: decision support tool.

## Discussion

### Principal Findings

In our study, DSTs increased laypersons’ accuracy and certainty in decision making. Potentially, DSTs can also reduce the mental effort in decision making, but this effect was statistically nonsignificant in our experiment. Thus, our experiment confirms the benefit of DSTs for COVID-19-related self-triage discussed in prior studies [4,23].

Regarding the question of whether 1 mode of presentation of DSTs (ie, static or interactive presentation) is superior to the other, our experiment produced evidence that differences in measures of the quality of decision support and participants’ perception of the tools are small. Interactive DSTs are potentially more convenient to use. Users of the interactive DST rated the mental effort as lower and the perceived ease of use as higher than the users of the static DST. However, these differences were not significant and did not translate into a higher level of trust, greater perceived usefulness, or greater intention to use the tool in the future. On the contrary, users rated the static tool more favorably on these measures than the interactive DST. Overall, the effect sizes for differences in these measures were low and statistically nonsignificant.

To the best of our knowledge, our study is among the first to directly compare the effectiveness of different modes of presentation on the quality of support received from web-based, patient-facing clinical DSTs. Our results are in line with findings from similar research in a different field of application: In their 2018 meta-analysis on the use of PtDAs to support decisions concerning prostate cancer screening, Baptista et al [27] concluded that web-based decision aids reduce decisional conflict compared to no decision aid, but no more than static, printed-out decision aids. Comparability between Baptista et al [27] and our study is limited, as web-based decision aids and their paper-based printed-out versions assessed by Baptista et al [27] do not directly match the mock conversational agent and the static flowchart we presented digitally in our study. However, both results suggest that for a decision scenario with clear-cut options, the mode of presentation with a DST has little to no effect on how helpful it is for its users.

### Practical Implications

First, our results underline the benefits of making DSTs available to laypersons for decisions with clear-cut options, such as those encountered in the COVID-19 pandemic, as both decisional accuracy and trust in the decision increased when laypersons were supported by both mock DSTs. Second, the quality of decision support provided by the static flowchart and the interactive tool did not differ significantly, and post hoc sensitivity analyses on effect sizes indicated that we can rule out large effects. Thus, factors such as development effort might potentially weigh more than the format of interaction when deciding on how to present decision support to laypersons.

When complexity is low, as in the case of the COVID-19 decisions, the static version may provide a better overview and thus make the decision more transparent to its users and be quicker and more cost-efficient to develop and publish than an interactive conversational agent. In contrast, for decisions with higher complexity, full transparency might compromise ease of use of a static DST. Thus, for more complex decisions, interactive tools that guide the user step-by-step may be more user friendly. However, our results suggest that interactivity is not an effective means in itself to increase the usefulness of a DST: First, the higher convenience of using the interactive DST did not translate into greater trust or perceived usefulness, nor did it yield a greater increase in decision accuracy or certainty than static flowcharts. As the latter entail less development efforts, while increasing transparency, they might be the preferable mode of DSTs for public health officials to implement on their websites to provide guidance to the public on decisions of low complexity and limited decision space.

Our study also raises 2 topics for further research. First, which factors increase users’ trust in DSTs, and how are they weighed? From our results, we can only speculate that users prefer transparency over convenience. Second, would subgroups of the population prefer 1 type of interface over the other? Although our results showed only minor differences between sample averages of users of the static and interactive DSTs, potentially subgroups of the population benefit more from 1 type of interface than the other. For example, users with low technological affinity might prefer a static flowchart over a conversational agent.

### Limitations

The results of our study are mainly limited by concerns about external validity. That is, the interactive mock DST we developed does not fully represent the whole variety of interactive tools that are available on the internet. Although our DST mimics the interactive tool from Johns Hopkins University, other tools incorporate significantly more decision factors and possible outcomes. Second, the sampled study population’s composition of educational background is not representative of the adult US population. Our study sample included a higher proportion of highly educated persons, with only 4 (2%) of 196 participants being nonhighschool graduates as compared to 9.8% in the US adult population [49]. In addition, the mock interactive tool we developed for this paper does not fully exhaust the potential of interactivity, as our participants only followed prompts to respond to binary questions by clicking either Yes or No buttons, while more interactive tools also require users to enter information manually (eg, age or current location). Finally, participants in this study did not use the tool for self-assessment but to appraise cases with fictitious patients. This means that they did not experience personal concerns, as would likely be the case with COVID-19 suspicion in a real-world use situation. To promote external validity, the recommendations of the DSTs we developed conform with all CDC guidelines and their interactive capacities are basic but mimic those of existing DSTs.

### Conclusion

When the decision space is limited, a static flowchart potentially performs just as well as an interactive tool in enhancing the decision quality of laypersons with symptoms indicative of a COVID-19 infection. As static flowcharts reveal their underlying decision algorithm more transparently, they might prove to be more suitable in not only guiding laypersons through the health care system but also communicating the reasoning and thereby empowering patients. Further research should validate our findings on different use cases, elaborate on the trade-off between transparency and convenience in DSTs, and investigate whether subgroups of users benefit more from 1 type of user interface than the other by assessing interactions between outcome variables (eg, accuracy, mental effort, perceived usefulness) and participant characteristics (eg, age, eHealth literacy). We have made all data necessary to conduct these exploratory analyses and to reproduce our reported findings publicly available [50].

## Appendix

Multimedia Appendix 1 Static decision support flowchart for laypersons with suspected COVID-19 infection developed as part of the scientific study.

Multimedia Appendix 2 Interactive decision support tool for laypersons with suspected COVID-19 infection developed as part of the scientific study.

Multimedia Appendix 3 List of COVID-19 symptoms according to the Robert Koch Institute.

Multimedia Appendix 4 List of risk factors for COVID-19 according to the Robert Koch Institute.

Multimedia Appendix 5 Overview of the case vignettes developed as part of the scientific study.

Multimedia Appendix 6 Outline of all 7 case vignettes developed as part of the scientific study.

Multimedia Appendix 7 Multiple-choice questions used to assess the participants' prior knowledge.

Multimedia Appendix 8 Decisions on each case vignette in the control group without decision support.

Multimedia Appendix 9 Decisions on each case vignette in the intervention groups with decision support, here illustrated by the flowchart example.

Multimedia Appendix 10 Single item for mental effort.

Multimedia Appendix 11 Decisional Conflict Subscale.

Multimedia Appendix 12 Items measuring participants’ perspectives on the 2 DSTs with items on ease of use, usefulness, trust, and future intention to use the DSTs. DST: decision support tool.

Multimedia Appendix 13 Boxplot for decision accuracy in regard to decision support type. Responses where participants confused "quarantine" and "isolation" were rated as wrong in this analysis.

Multimedia Appendix 14 CONSORT-eHEALTH checklist (V 1.6.1).

## Abbreviations

CDC: Centers for Disease Control and Prevention
DST: decision support tool
PtDA: patient decision aid
SC: symptom checker

## References

[1] Sohrabi C, Alsafi Z, O'Neill N, Khan M, Kerwan A, Al-Jabir A, Iosifidis C, Agha R (2020). World Health Organization declares global emergency: a review of the 2019 novel coronavirus (COVID-19). Int J Surg.

[2] World Health Organization Pandemie der Coronavirus-Krankheit (COVID-19).

[3] Epidemiologischer Steckbrief zu SARS-CoV-2 und COVID-19. Demografische Faktoren, Symptome und Krankheitsverlauf. Robert-Koch-Institut.

[4] Judson T, Odisho A, Neinstein A, Chao J, Williams A, Miller C, Moriarty T, Gleason N, Intinarelli G, Gonzales R (2020). Rapid design and implementation of an integrated patient self-triage and self-scheduling tool for COVID-19. J Am Med Inform Assoc.

[5] Schmieding ML, Mörgeli R, Schmieding MAL, Feufel MA, Balzer F (2021). Benchmarking triage capability of symptom checkers against that of medical laypersons: survey study. J Med Internet Res.

[6] What to Do if You Are Sick. Centers for Disease Control and Prevention.

[7] Coronavirus Disease 2019 (COVID-19) Risk Assessment and Public Health Management Decision Making. Centers for Disease Control and Prevention.

[8] COVID-19 Flowchart. Jackson Free Press.

[9] North Dakota Department of Health What To Do If You Are A Close Contact.

[10] COVID-19: Bin ich betroffen und was ist zu tun?: Orientierungshilfe für Bürgerinnen und Bürger. Robert-Koch-Institut.

[11] Contact Tracing Flow Chart: For potential and actual exposures to COVID-19. State of Michigan.

[12] Coronavirus-Entscheidungshilfe. ZEIT ONLINE GmbH.

[13] Ada Health GmBH COVID-19 Screener. 2020.

[14] Coronavirus Self-Checker. Centers for Disease Control and Prevention.

[15] CovApp: Haben Sie Symptome? CovApp zeigt Ihnen mögliche nächste Schritte. Charité – Universitätsmedizin Berlin.

[16] Emory University School of Medicine Coronavirus Checker.

[17] Together against Coronavirus: COVID-GUIDE. in4medicine AG.

[18] Coronavirus Self-Checker and COVID-19 Vaccine FAQ. The Johns Hopkins University, The Johns Hopkins Hospital, and The Johns Hopkins Health System Corporation.

[19] Universitätsklinikum Giessen und Marburg GmbH COVID-ONLINE.

[20] Covid Assessment. The USC Gehr Family Center for Health Systems Sciences and Innovation.

[21] COVID-19 Symptom Checker. WebMD LLC.

[22] Kahnbach L, Lehr D, Brandenburger J, Mallwitz T, Jent S, Hannibal S, Funk B, Janneck M (2021). Quality and adoption of COVID-19 tracing apps and recommendations for development: systematic interdisciplinary review of European apps. J Med Internet Res.

[23] Lunn PD, Timmons S, Julienne H, Belton CA, Barjaková M, Lavin C, McGowan FP (2021). Using decision aids to support self-isolation during the COVID-19 pandemic. Psychol Health.

[24] Munsch N, Martin A, Gruarin S, Nateqi J, Abdarahmane I, Weingartner-Ortner R, Knapp B (2020). Diagnostic accuracy of web-based COVID-19 symptom checkers: comparison study. J Med Internet Res.

[25] Perlman A, Vodonos Zilberg A, Bak P, Dreyfuss M, Leventer-Roberts M, Vurembrand Y, Jeffries HE, Fisher E, Steuerman Y, Namir Y, Goldschmidt Y, Souroujon D (2020). Characteristics and symptoms of app users seeking COVID-19-related digital health information and remote services: retrospective cohort study. J Med Internet Res.

[26] Zens M, Brammertz A, Herpich J, Südkamp N, Hinterseer M (2020). App-based tracking of self-reported COVID-19 symptoms: analysis of questionnaire data. J Med Internet Res.

[27] Baptista S, Teles Sampaio E, Heleno B, Azevedo LF, Martins C (2018). Web-based versus usual care and other formats of decision aids to support prostate cancer screening decisions: systematic review and meta-analysis. J Med Internet Res.

[28] If You Are Sick or Caring for Someone. Centers for Disease Control and Prevention.

[29] Microsoft Corporation Microsoft PowerPoint (Version 2019).

[30] InVision. InVisionApp Inc.

[31] (2021). Über das Online-Befragungstool UNIPARK. Trivian XI GmbH.

[32] Wessel D, Attig C, Franke T (2019). ATI-S: an ultra-short scale for assessing Affinity for Technology Interaction in user studies.

[33] Kim J, Park H (2012). Development of a health information technology acceptance model using consumers' health behavior intention. J Med Internet Res.

[34] Paas F, Tuovinen JE, Tabbers H, Van Gerven PWM (2003). Cognitive load measurement as a means to advance cognitive load theory. Educ Psychol.

[35] Kirschner P, Kirschner F, Seel NM (2012). Mental effort. Encyclopedia of the Sciences of Learning.

[36] O'Connor AM (2016). Validation of a Decisional Conflict Scale. Med Decis Making.

[37] Ammenwerth E, Scott P, de Keizer N, Georgiou A (2019). Technology acceptance models in health informatics: TAMUTAUT. Applied Interdisciplinary Theory in Health Informatics.

[38] Davis FD (1989). Perceived usefulness, perceived ease of use, and user acceptance of information technology. MIS Q.

[39] Online Participant Recruitment For Surveys and Market Research. Prolific Academic.

[40] Peer E, Brandimarte L, Samat S, Acquisti A (2017). Beyond the Turk: alternative platforms for crowdsourcing behavioral research. J Exp Soc Psychol.

[41] Rouse SV (2015). A reliability analysis of Mechanical Turk data. Comput Hum Behav.

[42] Casey LS, Chandler J, Levine AS, Proctor A, Strolovitch DZ (2017). Intertemporal differences among MTurk workers: time-based sample variations and implications for online data collection. SAGE Open.

[43] R Core Team (2021). R: A Language and Environment for Statistical Computing.

[44] Wickham H, Averick M, Bryan J, Chang W, McGowan L, François R, Grolemund G, Hayes A, Henry L, Hester J, Kuhn M, Pedersen T, Miller E, Bache S, Müller K, Ooms J, Robinson D, Seidel D, Spinu V, Takahashi K, Vaughan D, Wilke C, Woo K, Yutani H (2019). Welcome to the Tidyverse. JOSS.

[45] Wickham H (2021). ggplot2: 3.3.5.

[46] Delacre M, Lakens D, Leys C (2017). Why psychologists should by default use Welch’s t-test instead of Student’s t-test. Int Rev Soc Psychol.

[47] Kassambara A rstatix: Pipe-Friendly Framework for Basic Statistical Tests.

[48] Champely S pwr: Basic Functions for Power Analysis.

[49] United States Census Bureau Educational Attainment in the United States.

[50] Röbbelen A, Schmieding ML, Kopka M, Balzer F, Feufel MA (2021). Data set supplementing “Interactive versus static decision support tools for COVID-19: An experimental comparison. Zenodo.

