# Supplementary material for: Interactive Versus Static Decision Support Tools for COVID-19: Randomized Controlled Trial
Source: JMIR Public Health Surveill. 2022 Apr 15;8(4):e33733. doi: 10.2196/33733 (PMC9015012; doi:10.2196/33733)
Supplement: Multimedia Appendix 1 [file publichealth_v8i4e33733_app1.pdf]

JOHNS HOPKINS MEDICINE

Welcome to our Coronavirus Chart.

Are you experiencing a life-threatening emergency, such as severe shortness of breath or high fever?

Yes → **Call 911 or go to an emergency room.**

No ↓

Do you have any of the following?

- Cough
- Fever or chills
- Shortness of breath or difficulty breathing
- Muscle or body aches
- Sore throat
- New loss of taste or smell
- Diarrhea
- Headache
- Nausea or vomiting
- New fatigue
- Congestion or runny nose

Yes →

Do any of the following risk factors apply to you?

- High age (65 years or older)
- Smoking
- Pregnancy
- Preexisting conditions:
  - Chronic lung disease, such as moderate to severe asthma, Chronic obstructive pulmonary disease, cystic fibrosis, or pulmonary fibrosis
  - Serious heart condition, such as heart failure, coronary artery disease, or cardiomyopathy
  - Weakened immune system or taking medications that may cause immune suppression
  - Obesity
  - Diabetes, chronic kidney disease, or liver disease
  - High blood pressure
  - Blood disorder, such as sickle cell disease or thalassemia
  - Cerebrovascular disease or neurologic condition, such as stroke or dementia

No ↓

Isolation
Stay home at least 10 days since symptoms first appeared.

At this time it is not necessary to contact your healthcare provider. If you feel worse or think that it is an emergency, seek medical care immediately.

Yes ↓

Isolation
Stay home except to get medical care at least 10 days since symptoms first appeared.

You should call your healthcare provider and describe your symptoms.

No ↓ (from "Do you have any of the following?")

Have you had close contact with someone diagnosed with COVID-19 or been notified that you may have been exposed to it?

No ↓

You are at low risk for COVID-19 at this time. It is not necessary to contact your healthcare provider.

However, you should protect yourself and others from the spread of COVID-19.
Follow the current hygiene recommendations, incl. **keeping physical distance** to others.

Yes ↓

Quarantine
Stay home for 14 days and monitor for symptoms of COVID-19.

At this time it is not necessary to contact your healthcare provider.
