# Supplementary material for: Interactive Versus Static Decision Support Tools for COVID-19: Randomized Controlled Trial
Source: JMIR Public Health Surveill. 2022 Apr 15;8(4):e33733. doi: 10.2196/33733 (PMC9015012; doi:10.2196/33733)
Supplement: Multimedia Appendix 2 [file publichealth_v8i4e33733_app2.pdf]

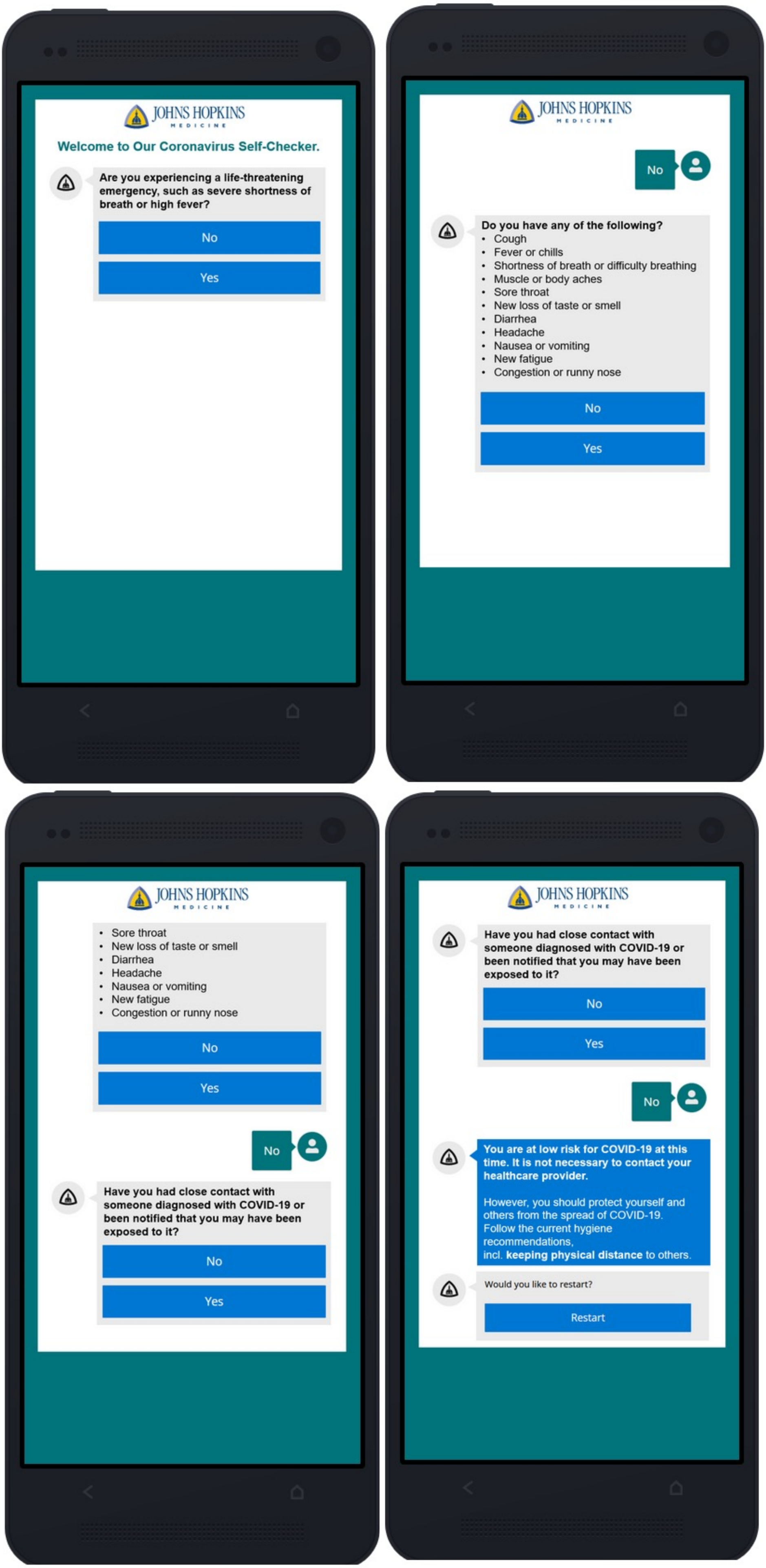
JOHNS HOPKINS MEDICINE
Welcome to Our Coronavirus Self-Checker.
Are you experiencing a life-threatening emergency, such as severe shortness of breath or high fever?
No
Yes
JOHNS HOPKINS MEDICINE
No
Do you have any of the following?
• Cough
• Fever or chills
• Shortness of breath or difficulty breathing
• Muscle or body aches
• Sore throat
• New loss of taste or smell
• Diarrhea
• Headache
• Nausea or vomiting
• New fatigue
• Congestion or runny nose
No
Yes
JOHNS HOPKINS MEDICINE
• Sore throat
• New loss of taste or smell
• Diarrhea
• Headache
• Nausea or vomiting
• New fatigue
• Congestion or runny nose
No
Yes
No
Have you had close contact with someone diagnosed with COVID-19 or been notified that you may have been exposed to it?
No
Yes
JOHNS HOPKINS MEDICINE
Have you had close contact with someone diagnosed with COVID-19 or been notified that you may have been exposed to it?
No
Yes
No
You are at low risk for COVID-19 at this time. It is not necessary to contact your healthcare provider.
However, you should protect yourself and others from the spread of COVID-19. Follow the current hygiene recommendations, incl. keeping physical distance to others.
Would you like to restart?
Restart
