# Supplementary material for: Interactive Versus Static Decision Support Tools for COVID-19: Randomized Controlled Trial
Source: JMIR Public Health Surveill. 2022 Apr 15;8(4):e33733. doi: 10.2196/33733 (PMC9015012; doi:10.2196/33733)
Supplement: Multimedia Appendix 3 [file publichealth_v8i4e33733_app3.docx]

| **Recorded symptoms for COVID-19 cases according to RKI,** (here defined as primary symptoms): |
| --- |
| - Cough |
| - Sneezing |
| - Fever |
| - Disturbance of sense of smell and taste |
| - Pneumonia |
| **Other symptoms according to RKI** (here defined as secondary symptoms): |
| Sore throat, shortness of breath, headache, limb pain, loss of appetite, weight loss, nausea, abdominal pain, vomiting, diarrhea, conjunctivitis, rash, lymph node swelling, apathy, somnolence |
