# Supplementary material for: Interactive Versus Static Decision Support Tools for COVID-19: Randomized Controlled Trial
Source: JMIR Public Health Surveill. 2022 Apr 15;8(4):e33733. doi: 10.2196/33733 (PMC9015012; doi:10.2196/33733)
Supplement: Multimedia Appendix 4 [file publichealth_v8i4e33733_app4.docx]

| **Risk groups for severe courses of COVID-19 disease according to RKI:** |
| --- |
| - Elderly persons (with steadily increasing risk of severe course from about 50-60 years of age) - Male gender - Smoker - Obese (BMI>30) and severely obese (BMI>35) people - People with Down syndrome (trisomy 21) - People with certain pre-existing conditions, without ranking: - Of the cardiovascular system (e.g., coronary artery disease and hypertension) - chronic lung disease (e.g., COPD) - chronic kidney and liver diseases - patients with diabetes mellitus (diabetes) - patients with cancer - patients with weakened immune system |
