# Supplementary material for: Interactive Versus Static Decision Support Tools for COVID-19: Randomized Controlled Trial
Source: JMIR Public Health Surveill. 2022 Apr 15;8(4):e33733. doi: 10.2196/33733 (PMC9015012; doi:10.2196/33733)
Supplement: Multimedia Appendix 5 [file publichealth_v8i4e33733_app5.pdf]

Overview of the case vignettes

Case vignette	Symptom	Contact situation	Risk factor	Recommendation on help-seeking behavior	Recommendation on social behavior
Harold	No symptoms	No close contact	Smoker	Selfcare (3)	Physical Distancing (1)
Mia	symptoms	No close contact	Chronic lung disease	Non-Emergency care (2)	Isolation (3)
Abigail	symptoms	Potential close contact	Smoker	Non-Emergency care (2)	Isolation (3)
Isabella	No symptoms	Close contact	none	Selfcare (3)	Quarantine (2)
Robert	symptoms	No close contact	none	Selfcare (3)	Isolation (3)
Laura	Emergency symptoms	Close contact	none	Emergency care (1)	Emergency care: quarantine or isolation is inappropriate (4)
William	Emergency symptoms	No close contact	High age	Emergency care (1)	Emergency care: quarantine or isolation is inappropriate (4)

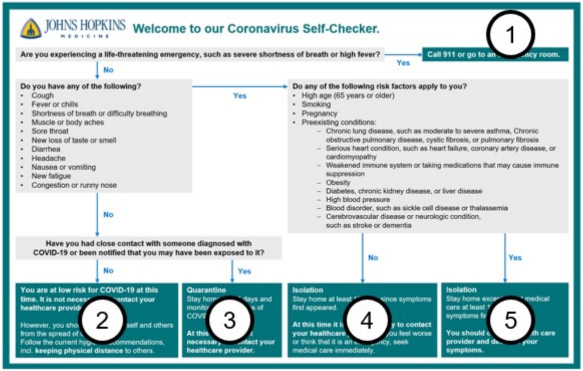

Possible outcomes:

1) Emergency (Laura, William)
2) Selfcare and physical distancing (Harold)
3) Selfcare and quarantine (Isabella)
4) Selfcare and isolation (Robert)
5) Non-Emergency care and isolation (Mia, Abigail)
