# Supplementary material for: Interactive Versus Static Decision Support Tools for COVID-19: Randomized Controlled Trial
Source: JMIR Public Health Surveill. 2022 Apr 15;8(4):e33733. doi: 10.2196/33733 (PMC9015012; doi:10.2196/33733)
Supplement: Multimedia Appendix 6 [file publichealth_v8i4e33733_app6.docx]

| Helen, 60 years old  - Helen lives with her son. They have reduced their contact with other people as much as possible. - Her son has been suffering from flu-like symptoms since yesterday and has not been in the same room with Helen since. He has also done a COVID 19 test directly, but the result is not yet attention check for all questions below select the second option (from the top). - Today Helen feels a severe and constant pain and pressure in the chest. She also has difficulty breathing. - She has a weakened immune system due to taking medications that cause immune suppression. But apart from that she is in good health. - She is a non-smoker. |
| --- |
| Harold, 53 years old  - Harold works as a policeman and so despite the spread of COVID-19 he often has contact with different people. However, of course he tries to keep the rules of hygiene in the best possible way. - This morning he received the news that a colleague had tested positive for COVID-19. He did not have any close contact with this colleague, as they work in different departments. - He has no previous illnesses and is in good health. - He is a non-smoker. - The last time he was abroad was one year ago. |
| Mia, 30 years old  - Mia works as a teacher in a primary school. - She pays special attention to her health and tries to implement all hygiene measures conscientiously. Yesterday one of the students called in sick. The student is getting tested for COVID-19, but has not yet received a test result. Mia has not had any close contact with the student. - This morning she woke up with a slight scratching of her throat and body aches and felt a little weak. - She has suffered from severe asthma since childhood. She's a non-smoker. - She is not pregnant. - The last time she was abroad was in November last year. |
| Abigail, 53 years old  - Abigail has been working in the home office since the spread of COVID-19. - Since the day before yesterday she feels a bit fatigued. When she took her temperature today, she noticed that she had a moderate fever of 38,8 Celsius/ 101.84 °F. In addition, she now also suffers from a dry cough. - Five days ago, it was Abigail's birthday and she met up with a friend. The friend coughed from time to time that evening, but otherwise seemed healthy. She hasn't heard anything from this person after the get-together, so she doesn’t know whether he tested positive for COVID-19. - She has no previous illnesses. She has been smoking since early adulthood. - The last time she was abroad was last year. |
| Isabella, 49 years old  - Isabella lives alone, but visits her parents daily to help them with physically difficult tasks. They are all in good health. - She works in the administration of a shopping centre and is responsible for the coordination of the employees. - Today a colleague called her to tell her that he is sick and infected with COVID-19. Yesterday she had worked with this colleague. It occurs to her that she had close contact with him because they were sitting next to each other at lunch. - She has no previous illnesses and is a non-smoker. - The last time she went abroad was more than a year ago. - She is not pregnant. |
| Robert, 54 years old  - Robert works for a business consultancy and has been in the home office since the spread of COVID-19. Therefore, he has only little contact to other people. - Since yesterday Robert suffers from a dry cough. Today he has also developed a moderate fever of 38,5 degrees Celsius/ 101.3 ° F. - He has no previous illnesses and is otherwise in good health. He's a non-smoker. - The last trip abroad by him and his family was more than a year ago. |
| Laura, 25 years old  - Laura lives together with three friends in a shared apartment. - One of her friends works at the hospital. It came out today that she got infected with COVID-19 while seeing a patient. In the meantime, Laura has been in close contact with her friend. - Upon waking up Laura noticed that she felt dizzy. Since the morning her temperature increased and now she has a high fever of 41 °C/ 105.8 °F. In the last hour she became drowsy and developed a headache. - She has no previous illnesses. She's a non-smoker. - She is not pregnant. |
| William, 80 years old  - William has retired. Since the spread of COVID-19, his wife and he have also severely restricted all further contacts. He only leaves the house to shop groceries. - During the last night he started to have difficulty breathing. Within the last hour the shortness of breath worsened. He starts sweating and gasping for air. - He has no previous illnesses. He is a non-smoker. - The last time he went abroad was more than three years ago. |
