# Supplementary material for: Interactive Versus Static Decision Support Tools for COVID-19: Randomized Controlled Trial
Source: JMIR Public Health Surveill. 2022 Apr 15;8(4):e33733. doi: 10.2196/33733 (PMC9015012; doi:10.2196/33733)
Supplement: Multimedia Appendix 7 [file publichealth_v8i4e33733_app7.pdf]

Five questions related to COVID-19

Please answer the following multiple choice questions by selecting the correct answer option(s).
For each question one, several or all of the answer options may be correct.

1. Which of these symptoms indicate a COVID-19 infection?

Please select all that apply.

- ☐ Shortness of breath
- ☐ Earache
- ☐ Dry cough
- ☐ Fever

2. In times of the spread of COVID-19, for which symptoms should you call 911 or go to the emergency room?

Please select all that apply.

- ☐ After you had close contact with a person infected with COVID-19, even though you are symptom-free
- ☐ In case of high fever at or above 105,8 °F / 41°C
- ☐ In case of severe breathing difficulties
- ☐ In case of dry cough

3. What should you do if you have worsening symptoms of a possible COVID-19 infection, but it is not an emergency?

Please select all that apply.

- ☐ Call your general practitioner
- ☐ Go immediately to your general practitioner
- ☐ Call 911
- ☐ Wait 14 days

4. What are risk factors for a severe course of COVID-19 infection?

Please select all that apply.

- ☐ Chronic health problems (such as diabetes, heart disease, ...)
- ☐ A young age (under 18 years)
- ☐ A high age (from 65 years upwards)
- ☐ A suppression of the immune system (such as people with HIV/AIDS and cancer)

5. In times of the spread of COVID-19, when should you go into quarantine?

Please select all that apply.

- ☐ If I have had close contact with a person who is confirmed to be infected with COVID-19 and has symptoms
- ☐ If I am confirmed to be infected with COVID-19 and have current symptoms
- ☐ After hearing that a distant friend (no recent physical contact) was infected with COVID-19
- ☐ In none of the situations listed
