# Supplementary material for: Interactive Versus Static Decision Support Tools for COVID-19: Randomized Controlled Trial
Source: JMIR Public Health Surveill. 2022 Apr 15;8(4):e33733. doi: 10.2196/33733 (PMC9015012; doi:10.2196/33733)
Supplement: Multimedia Appendix 8 [file publichealth_v8i4e33733_app8.pdf]

1. What do you think: what level of medical care is most appropriate for this person?

Please select the level of care you would recommend to this person.

- ◯ **Emergency care** - The person should call 911 or go to an emergency room.
- ◯ **Non-emergency care** - The person should call his/her healthcare provider, but calling 911 or going to an emergency room is *not* required.
- ◯ **No professional medical care** is required at this stage.

2. What do you think: is no extra measure, quarantine or self-isolation required?

Please select what you would recommend to this person. Note: Non-emergency care is still possible even in quarantine or isolation.

- ◯ **No extra measures** need to be taken, apart from following the current hygiene recommendations (incl. keeping physical distance to others).
- ◯ **Quarantine** is required.
- ◯ **Isolation** is required.
- ◯ *Not applicable, because the person should seek emergency care.*
