# Supplementary material for: Interactive Versus Static Decision Support Tools for COVID-19: Randomized Controlled Trial
Source: JMIR Public Health Surveill. 2022 Apr 15;8(4):e33733. doi: 10.2196/33733 (PMC9015012; doi:10.2196/33733)
Supplement: Multimedia Appendix 9 [file publichealth_v8i4e33733_app9.pdf]

1.1 According to the *flowchart*, what level of medical care is most appropriate for this person?

Please select the level of care that the flowchart recommends for this person.

- ○ **Emergency care** - The person should call 911 or go to an emergency room.
- ○ **Non-emergency care** - The person should call his/her healthcare provider, but calling 911 or going to an emergency room is *not* required.
- ○ **No professional medical care** is required at this stage.

1.2 What do you think: what level of medical care is most appropriate for this person?

Please select the most appropriate level of care for this person from your personal and purely medical point of view and without regard to financial considerations (e.g. how much a visit might cost).

- ○ **Emergency care** - The person should call 911 or go to an emergency room.
- ○ **Non-emergency care** - The person should call his/her healthcare provider, but calling 911 or going to an emergency room is *not* required.
- ○ **No professional medical care** is required at this stage.

2.1 According to the *flowchart*, is no extra measure, quarantine or self-isolation required?

Please select what the flowchart recommends for this person. Note: Non-emergency care is still possible even in quarantine or isolation.

- ○ **No extra measures** need to be taken, apart from following the current hygiene recommendations (incl. keeping physical distance to others).
- ○ **Quarantine** is required.
- ○ **Isolation** is required.
- ○ *Not applicable, because the person should seek emergency care.*

2.2 What do you think: is no extra measure, quarantine or self-isolation required?

Note: Non-emergency care is still possible even in quarantine or isolation.

- ○ **No extra measures** need to be taken, apart from following the current hygiene recommendations (incl. keeping physical distance to others).
- ○ **Quarantine** is required.
- ○ **Isolation** is required.
- ○ *Not applicable, because the person should seek emergency care.*
