# Supplementary material for: Interactive Versus Static Decision Support Tools for COVID-19: Randomized Controlled Trial
Source: JMIR Public Health Surveill. 2022 Apr 15;8(4):e33733. doi: 10.2196/33733 (PMC9015012; doi:10.2196/33733)
Supplement: Multimedia Appendix 10 [file publichealth_v8i4e33733_app10.pdf]

Indicate the amount of mental effort that you spent on evaluating the case you have just finished.

1 - Very, very low mental effort	2 - Very low	3 - Low	4 - Rather low	5 - Neither low nor high	6 - Rather high	7 - High	8 - Very high	9 - Very, very high mental effort
