# Supplementary material for: Interactive Versus Static Decision Support Tools for COVID-19: Randomized Controlled Trial
Source: JMIR Public Health Surveill. 2022 Apr 15;8(4):e33733. doi: 10.2196/33733 (PMC9015012; doi:10.2196/33733)
Supplement: Multimedia Appendix 11 [file publichealth_v8i4e33733_app11.pdf]

Now, thinking about the decisions you just made, please look at the following comments.

Please, show how strongly you agree or disagree with these comments.

	Strongly disagree	Disagree	Neither agree nor disagree	Agree	Strongly agree
I was clear about the best choices.	○	○	○	○	○
I felt sure about what to choose.	○	○	○	○	○
Select 'Disagree' for this item.	○	○	○	○	○
These decisions were easy for me to make.	○	○	○	○	○
