# Supplementary material for: Interactive Versus Static Decision Support Tools for COVID-19: Randomized Controlled Trial
Source: JMIR Public Health Surveill. 2022 Apr 15;8(4):e33733. doi: 10.2196/33733 (PMC9015012; doi:10.2196/33733)
Supplement: Multimedia Appendix 12 [file publichealth_v8i4e33733_app12.pdf]

Usefulness and Ease of Use of the flowchart

Think of the flowchart that was made available to you to help you make decisions.

Ease of Use

For the following statements, select the box that best describes your reaction to the flowchart.

	1 - Strongly disagree	2	3	4	5 - Strongly agree
My interaction with the flowchart is clear and understandable.	○	○	○	○	○
Interacting with the flowchart does not require a lot of my mental effort.	○	○	○	○	○
I find the flowchart to be easy to use.	○	○	○	○	○
I find it easy to get the flowchart to do what I want it to do.	○	○	○	○	○

Usefulness

For the following statements, select the box that best describes your reaction to the flowchart.

	1 - Strongly disagree	2	3	4	5 - Strongly agree
Using the flowchart improves my performance in my decision.	○	○	○	○	○
Using the flowchart in my decision increases my productivity.	○	○	○	○	○
Using the flowchart enhances my effectiveness in my decision.	○	○	○	○	○
I find the flowchart to be useful in my decision.	○	○	○	○	○

How much did you trust the recommendations of the flowchart?

Please rate your trust in the flowchart on the following scale.

1 - Not at all | 2 | 3 | 4 | 5 | 6 | 7 - Completely

How likely is it that you would use the flowchart if you were faced with a similar decision?

Please indicate on the following scale how likely it is that you would use the flowchart if it were available to you.

1 - Highly unlikely | 2 | 3 | 4 | 5 | 6 | 7 - Highly likely
