# Supplementary figures and images for: Interactive Versus Static Decision Support Tools for COVID-19: Randomized Controlled Trial
Source: JMIR Public Health Surveill. 2022 Apr 15;8(4):e33733. doi: 10.2196/33733 (PMC9015012; doi:10.2196/33733)

**Decision accuracy in regard to decision support type**

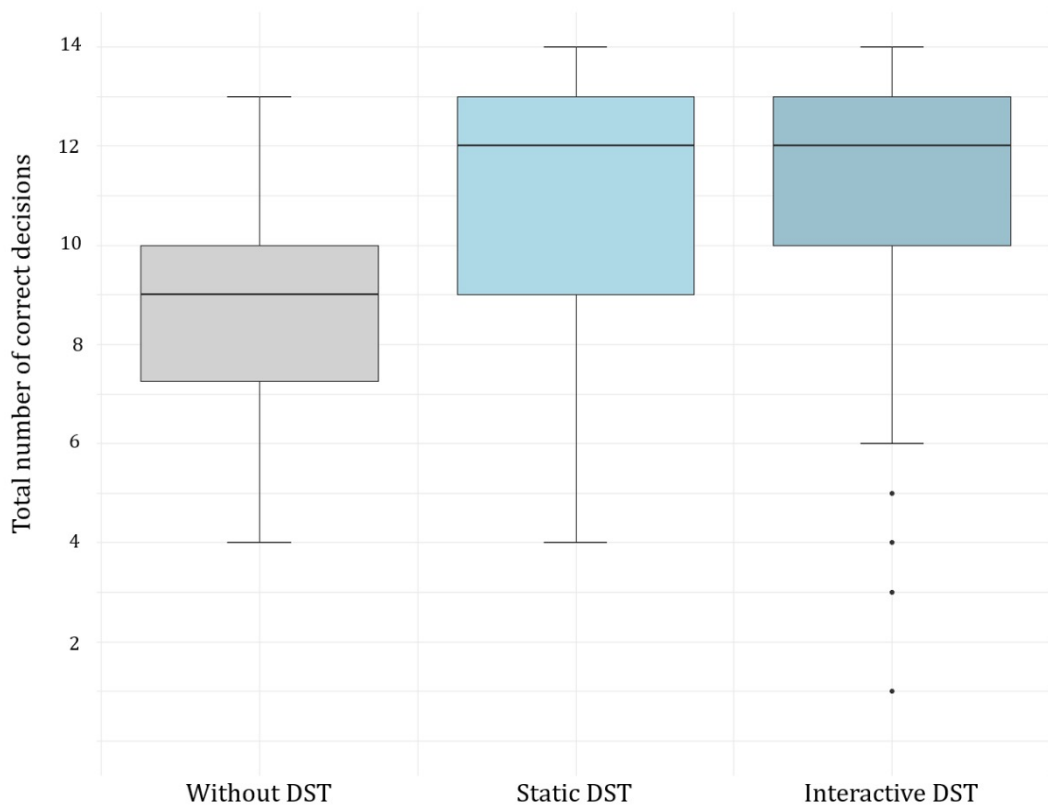

Supplement: Multimedia Appendix 13 [file publichealth_v8i4e33733_app13.pdf]
